# Editorial: Exploring small molecule inhibitors in cardiovascular and cerebrovascular diseases

**DOI:** 10.3389/fphar.2025.1559488

**Published:** 2025-02-10

**Authors:** Basveshwar Gawali, Shweta Shrivastava, R. Uday Kumar, Satyendra Kumar Rajput, Manish Kumar Jeengar

**Affiliations:** ^1^ Department of Radiation Oncology, SUNY Upstate Medical University, Syracuse, NY, United States; ^2^ School of Pharmacy, School of Health and Allied Sciences, ARKA JAIN University, Jamshedpur, Jharkhand, India; ^3^ Department of Pharmacy Practice, Amrita School of Pharmacy, Amrita Vishwa Vidyapeetham, Kochi, Kerala, India; ^4^ Department of Pharmaceutical Sciences, Gurukula Kangri Deemed to be University, Haridwar, Uttarakhand, India; ^5^ Department of Pharmacology, Amrita School of Pharmacy, Amrita Vishwa Vidyapeetham, Kochi, Kerala, India

**Keywords:** small molecule inhibitors, cardiovascular diseases, PDE4 inhibitors, NLRP3 inflammasome, non-invasive biomarkers, intracranial aneurysms, cerebrovascular diseases

## Introduction

Cardiovascular and cerebrovascular diseases remain leading causes of morbidity and mortality worldwide. The complex pathophysiology underlying these conditions necessitates innovative therapeutic approaches. Small molecule inhibitors (SMIs) have emerged as promising candidates, offering precise modulation of key molecular pathways involved in these diseases. This Research Topic presents a collection of insightful articles that delve into the mechanistic roles and therapeutic potential of SMIs, emphasizing their impact on inflammation, vascular remodeling, and cellular signaling in cardiovascular and cerebrovascular pathologies.

## Phosphodiesterase inhibitors: bridging inflammation and vascular protection

The mini-review by Fan et al. highlights the anti-inflammatory mechanisms of phosphodiesterase (PDE4) inhibitors, including their potential applications in vascular diseases. While clinically established for conditions like chronic obstructive pulmonary disease and psoriatic arthritis, this article underscores the unexplored role of PDE4 inhibitors in mitigating vascular inflammation. The authors emphasize the necessity for further investigation into the therapeutic efficacy and safety of these inhibitors in cardiovascular settings, advocating for the development of next-generation PDE4 inhibitors with minimized adverse effects.

## NLRP3 inflammasome: a target for atherosclerosis therapy

Chen and Li review provides a comprehensive analysis of the NLRP3 inflammasome’s role in atherosclerosis. This inflammasome, a critical mediator of vascular inflammation, contributes to endothelial injury, foam cell formation, and pyroptosis. The authors discuss emerging pharmacological strategies targeting the NLRP3 inflammasome, including small molecule inhibitors, and propose their integration into therapeutic regimens for atherosclerosis. By elucidating the molecular mechanisms, this article sets the stage for designing effective NLRP3-targeted therapies.

## Noninvasive biomarkers for cardiovascular disorders

The original research by Pulukool et al. explores noninvasive cardiac-specific biomarkers, such as high-sensitivity troponin I (hs-TnI) and high-sensitivity C-reactive protein (hs-CRP), in the diagnosis and prevention of vascular stenosis. This study not only identifies correlations between biomarker levels and stenosis severity but also highlights their utility in early risk stratification. By focusing on noninvasive diagnostics, the authors bridge the gap between molecular insights and clinical applications, emphasizing the potential of biomarkers in guiding personalized therapy.

## Advancing neuroprotection in intracranial aneurysms

Balkrishna et al. examine the application of SMIs in intracranial aneurysms (IAs), a condition characterized by high mortality risk due to rupture. Their review highlights the therapeutic roles of statins, matrix metalloproteinase inhibitors, and cytokine modulators in stabilizing aneurysm walls and reducing rupture risk. The authors advocate for targeted delivery systems to enhance the efficacy of SMIs while preserving healthy brain tissue. This article underscores the translational potential of SMIs in managing IAs, paving the way for future clinical trials.

## Insights from bibliometric analysis of SGLT2 inhibitors

The bibliometric analysis by Pan et al. offers a macroscopic view of the scientific landscape surrounding sodium-glucose co-transporter (SGLT2) inhibitors in cardiovascular diseases. By analyzing publication trends and research hotspots, the authors identify key areas of focus, such as the inhibitors’ cardioprotective effects. This article serves as a valuable resource for researchers, guiding future studies on the mechanistic and clinical implications of SGLT2 inhibitors.

The original research by Liu et al. investigates the regulatory role of transglutaminase in endothelial cell calcification through IL--mediated autophagy. The study highlights a novel pathway linking inflammation and calcification, proposing potential therapeutic interventions targeting this axis. By advancing our understanding of endothelial dysfunction, this work provides a foundation for developing targeted therapies in vascular calcification.

## Summary of contributions and table explanation

Table 1 below groups the articles included in this Research Topic according to their focus areas and total views as of 7 January 2025. This table serves as an overview of the diverse contributions, highlighting their main findings and relevance to the field of small molecule inhibitors.

**TABLE 1:** Summary of contributions.

Article name	Focus area	Key findings	Total views (as of 7 January 2025)	Article reference
PDE4 inhibitors: potential protective effects in inflammation and vascular diseases	PDE4 inhibitors	Anti-inflammatory mechanisms and vascular potential	2,554	Fan et al.
NLRP3 inflammasome in atherosclerosis: Mechanisms and targeted therapies	NLRP3 inflammasome in atherosclerosis	Targets and strategies for inflammasome inhibition	1,693	Chen and Li
Noninvasive cardiac-specific biomarkers for the diagnosis and prevention of vascular stenosis in cardiovascular disorder	Noninvasive biomarkers	hs-TnI and hs-CRP as diagnostic tools	1,040	Pulukool et al.
Small molecule inhibitors target multiple neuropathological signaling to exert novel neuroprotection in intracranial aneurysms	Intracranial aneurysms	Statins and cytokine modulators for neuroprotection	697	Balkrishna et al.
Bibliometric and visual analysis of SGLT2 inhibitors in cardiovascular diseases	SGLT2 inhibitors	Cardioprotective effects and publication trends	637	Zhang et al.
Transglutaminase 2 regulates endothelial cell calcification via IL-6-mediated autophagy	Endothelial dysfunction	Transglutaminase regulation of calcification via autophagy	351	Li et al.

## Concluding remarks and future directions

This Research Topic underscores the transformative potential of SMIs in addressing unmet clinical needs in cardiovascular and cerebrovascular diseases. The contributions span a diverse range of mechanisms, from modulating inflammation and calcification to enhancing neuroprotection and diagnostic accuracy. Moving forward, multidisciplinary collaborations are essential to translate these findings into clinical practice. Future research should prioritize optimizing the specificity, safety, and delivery of SMIs, ensuring their integration into personalized medicine. The editors extend their gratitude to the contributing authors, reviewers, and the editorial team for their invaluable efforts in advancing this dynamic field. We hope this Research Topic inspires further exploration and innovation in the development of small molecule-based therapies.

